# CT Radiomic Features of the Crystalline Lens and Association with Age, Hypertension and Cerebral White Matter Lesions

**DOI:** 10.3390/diagnostics16050763

**Published:** 2026-03-04

**Authors:** Anne Strübing, Estelle Akl, Chris Lappe, Stefan Polei, Oliver Stachs, Tobias Lindner, Mathias Manzke, Sönke Langner, Felix G. Meinel, Marc-André Weber, Thoralf Niendorf, Ebba Beller

**Affiliations:** 1 Institute of Diagnostic and Interventional Radiology, Pediatric Radiology and Neuroradiology, University Medical Center Rostock, 18057 Rostock, Germany; anne.struebing@uni-rostock.de (A.S.); estelle.akl@med.uni-rostock.de (E.A.); chris.lappe@med.uni-rostock.de (C.L.); stefan.polei@med.uni-rostock.de (S.P.); tobias.lindner@med.uni-rostock.de (T.L.); mathias.manzke@med.uni-rostock.de (M.M.); soenke.langner@med.uni-rostock.de (S.L.); felix.meinel@med.uni-rostock.de (F.G.M.); marc-andre.weber@med.uni-rostock.de (M.-A.W.); 2 Department of Ophthalmology, University Medical Center Rostock, 18057 Rostock, Germany; oliver.stachs@uni-rostock.de (O.S.); thoralf.niendorf@mdc-berlin.de (T.N.); 3 Berlin Ultrahigh Field Facility (B.U.F.F.), Max Delbrück Center for Molecular Medicine in the Helmholtz Association, 13125 Berlin, Germany; 4 Department Life, Light & Matter, University of Rostock, 18055 Rostock, Germany; 5 Core Facility Multimodal Small Animal Imaging, University Medical Center Rostock, 18057 Rostock, Germany

**Keywords:** radiomics, eye lens, computed tomography, white matter lesion, hypertension

## Abstract

**Background:** Radiomic analyses have been extensively explored in oncologic imaging and more recently in neuroimaging. However, radiomic characterization of the crystalline lens using computed tomography has not yet been systematically investigated. **Methods:** In this retrospective study, semiautomatic segmentation of the eye lens on orbital CT was performed on 112 patients (mean age 48 ± 20 years, 38% female). After radiomics feature extraction, a Boruta feature selection approach based on the random forest algorithm was applied to select the most relevant radiomics features. Severity of white matter lesions were graded according to the Fazekas scale for each patient on axial non-contrast head CT. **Results:** In total, 17 important features were associated with age-related changes in the eye lens and three important radiomic features for the differentiation between patients with a Fazekas score > 1 and a control group. Significantly higher values were found in patients with a Fazekas score > 1 compared to the control group regarding all three features, “ClusterShade”, “Skewness” and “DifferenceVariance” (*p* = 0.0006, 0.0023 and 0.0376, respectively), which are all measures of heterogeneity. No important radiomic features of the eye lens were confirmed between patients with and without hypertension. **Conclusions:** To the best of our knowledge, this is the first study to use CT-based radiomic analysis of the crystalline lens to detect differences among demographic or clinical groups with small vessel disease. The present results might help to expand the range of applications of radiomics regarding ophthalmic (patho-)physiology and suggest a possible new biomarker for systemic vascular diseases.

## 1. Introduction

Orbital computed tomography (CT) is commonly employed as a diagnostic modality, particularly for evaluating traumatic injuries in emergency situations [1]. As part of these examinations, the eye lens is captured on orbital CT images, yet they are rarely evaluated in detail [2]. Radiomics enables the derivation of numerous quantitative parameters that describe tissue intensities, texture patterns, and spatial heterogeneity. This methodology has demonstrated value in oncologic imaging and has increasingly been applied to neurologic diseases, where it can capture subtle microstructural changes that are not readily apparent on visual inspection [3,4,5,6,7]. Despite these advances, the application of radiomics to ocular structures—particularly the crystalline lens—remains largely unexplored.

The human lens undergoes continuous aging-related changes, including protein aggregation, fiber compaction, and increased light scattering, which are thought to contribute to lens opacification [2]. Furthermore, several studies have suggested associations between systemic vascular health and ocular structures, implicating the lens as a potential indicator of systemic aging or vascular disease [6,7,8]. White matter lesions of the brain are a manifestation of small vessel disease and frequently graded using the Fazekas scale [9]. Increased lesion burden has been linked to age, hypertension, cognitive decline, and increased risk of stroke [10]. Given the shared pathophysiological pathways involving microangiopathy and vascular disease, as well as the systemic effect on other tissues, it is plausible that they may also be reflected in the crystalline lens [11,12].

Therefore, the aim of this study was to determine whether aging, hypertension, and cerebral WML impact radiomic features extracted from orbital CT of the eye lens. This kind of advanced imaging analysis of the crystalline lens may provide further understanding of age-related changes in the lens and help to identify potential imaging biomarkers of systemic vascular disease.

## 2. Methods

### 2.1. Patient Selection and Study Design

This is a retrospective, single-center study. The research protocol received approval from the Ethics Committee of Rostock University Medical Faculty (blinded) which granted a waiver of informed consent. All procedures were carried out in accordance with the current version of the Declaration of Helsinki. Patients were selected through a retrospective review of our radiology information system database (Centricity 5.0, GE Healthcare, Barrington, IL, USA). Inclusion criteria were patients who received a non-contrast orbital CT scan at our institution between 1 March 2022 and 31 March 2024 (*n* = 414). We excluded patients with incomplete depiction of both eye lenses, bilateral artificial lenses, bilateral artifacts of the orbit or no additional cranial CT. The remaining patients were divided into three groups as follows: patients with no hypertension and Fazekas score ≤ 1 as control group, patients with Fazekas score > 1 (Fazekas cohort) and patients with hypertension (hypertension cohort). The left eye was used for evaluation unless it had to be excluded, e.g., due to artifacts, artificial lenses or if a periorbital hematoma was detected to rule out posttraumatic changes in the lens, in which case the right eye was analyzed instead.

### 2.2. CT Acquisition Protocol

The included patients underwent non-contrast orbital CT imaging and additional non-contrast head CT, using a 128-slice CT scanner (Revolution CT, GE Healthcare). Each patient was scanned in a supine position craniocaudally from the frontal sinus to the inferior boundary of the nasal cavity. The imaging protocol consisted of a slice thickness of 0.625 mm with 3.0 mm reconstructions, a tube voltage of 120 kV, and tube current modulation in mA determined from the scout acquisition.

### 2.3. Assessment of White Matter Lesions

**Figure 1:**
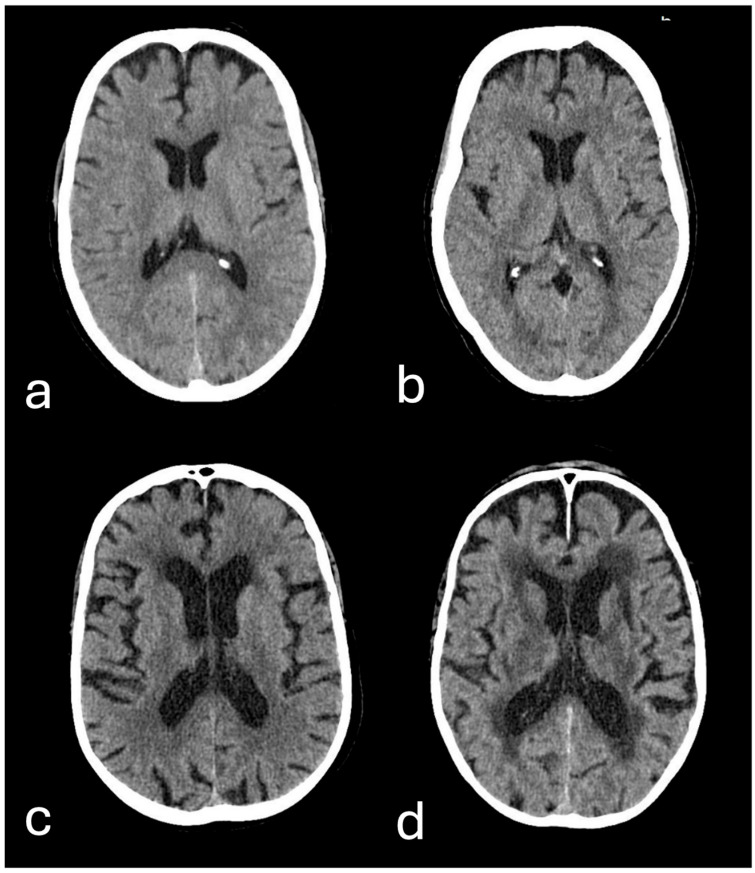
Axial non-contrast head CT images illustrating the categorization of white matter lesions according to the four-stage Fazekas scale: (**a**) normal brain (Fazekas 0), compared with (**b**) punctate lesions (Fazekas 1), (**c**) early confluent lesions (Fazekas 2), and (**d**) diffuse confluent lesions (Fazekas 3).

An independent review was performed on all axial non-contrast head CT scans by two radiologists with 3 and 11 years of clinical experience, respectively. The presence and severity of white matter lesions was rated according to the established Fazekas scale (grade 0, 1, 2 and 3) for each patient (Figure 1) [13,14]. Following the initial evaluation, any differences in Fazekas scores were recorded and reviewed by both readers. Subsequently, a consensus Fazekas score was determined for each patient.

### 2.4. Semiautomatic Segmentation of the Lens and Feature Extraction

**Figure 2:**
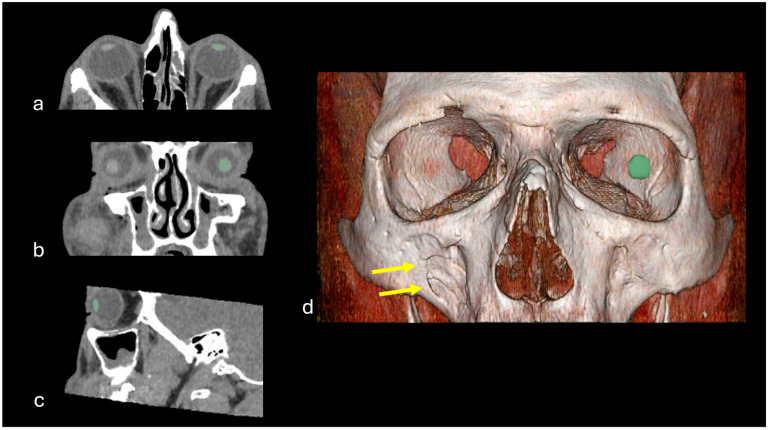
Representative orbital CT images (axial (**a**), coronal (**b**) and sagittal planes (**c**)) as well as reconstructed 3D model (**d**) of a 42-year-old patient from the control group, visiting the emergency room after a physical assault (hit to the face with a bottle) to rule out possible trauma. Clinically, there was swelling of the right cheek. CT imaging revealed an impression fracture of the ventral wall of the right maxillary sinus (yellow arrows). Lens segmentations performed with 3D Slicer are marked in green.

Semiautomatic segmentation of the crystalline lens was performed using the open-source software 3D Slicer (http://www.slicer.org, accessed on 30 May 2024, version 5.6.2,) and the Segment Editor module within 3D Slicer. For the segmentation process, the eye lens was identified manually, and a volume of interest was delineated by the software utilizing a region-growing algorithm. If necessary, manual corrections were applied by a radiologist (with 11 years of experience (E.B.)) to the contours obtained by the software. Figure 2 displays an example of semiautomatic segmentation of the eye lens. We extracted radiomics features by using the “SlicerRadiomics” extension which provides an interface to the Pyradiomics library (version 3.1.0), resulting in a sum of 97 radiomic features (first order statistics, GLCM, GLSZM, GLRLM, NGTDM and GLDM).

### 2.5. Statistical Analysis

To select the most relevant radiomic features, the established Boruta package was applied in RStudio (version 2025.12.1) using a random forest-based feature selection approach. The Boruta algorithm functions as a wrapper for a random forest classifier, aiming to determine the most significant variables within a dataset. Various model-building approaches have been compared by recent studies and showed that the random forest classification approach demonstrated the greatest predictive accuracy [15]. Details of feature selection with the Boruta package have previously been described elsewhere [16]. The variables’ importance scores of all radiomics features were calculated for age, Fazekas score > 1, and hypertension and are presented in the graphs. Blue boxplots depict the minimum, mean, and maximum Z scores of shadow attributes. Features that were rejected are shown in red, while those that were confirmed are displayed in green boxplots. Following feature selection, all variables identified as important were analyzed with GraphPad Prism, version 9.0.0 (GraphPad Software LLC, Boston, MA, USA), which included Pearson’s correlation coefficient (r) to evaluate the relationship between radiomics features and age and the Mann–Whitney U test was used to compare the confirmed features across the two groups (Fazekas score > 1 vs. age-matched control group). Differences were considered statistically significant if the *p*-value was less than 0.05.

## 3. Results

### 3.1. Patient Characteristics

The final study population included 112 patients with a mean age of 48 ± 20 years and 38% (*n* = 43) were female. They were subdivided into a control cohort with 82 subjects, a Fazekas cohort (with a Fazekas score > 1) with 17 subjects and a hypertension cohort with 17 subjects. There were four patients with overlap (both Fazekas score >1 as well as hypertension, mean age of 70 ± 17 years and 50% (*n* = 2) were female), which were assigned to both cohorts. Age-matched groups were formed from 17 subjects of the control cohort for the Fazekas and hypertension cohort, with a mean age of 72 ± 6 and 59 ± 17 years, respectively. The most common indication for obtaining a CT examination of the orbit was trauma (*n* = 105), followed by suspicion of foreign body inclusion (*n* = 2), postoperative control (*n* = 2), surgery planning (*n* = 1), inflammatory focus (*n* = 1) and retrobulbar mass (*n* = 1). The findings are summarized in Table 1.

### 3.2. Age-Related Changes in Radiomic Features of the Eye Lens Texture

**Figure 3:**
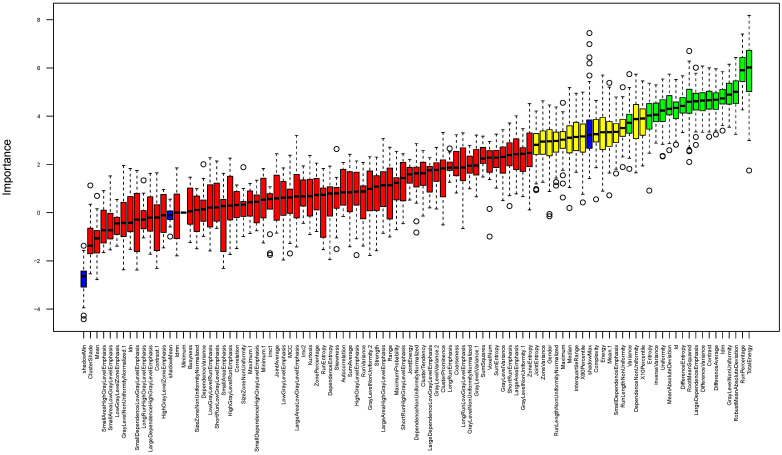
Selection of relevant features by using the random forest based Boruta algorithm of age-related changes based on the eye lens in CT with confirmed features highlighted in green, tentative features in yellow, features that were rejected in red and “shadow” features are shown in blue. The Boruta algorithm evaluates feature importance by comparing each real feature to so-called shadow features—randomly permuted copies of the original variables. Only features that reliably outperform these shadow features are considered relevant, ensuring robust selection in high-dimensional radiomics datasets. Circles represent outliers, while the whiskers denote the minimum and maximum values.

**Figure 4:**
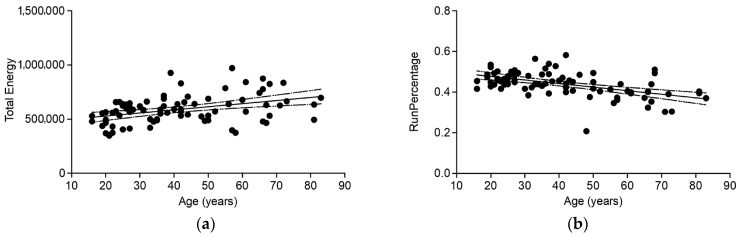
Scatterplots for the two most important features associated with age-related changes in the lens in CT ((**a**): “Total Energy” and (**b**): “RunPercentage”). The regression is shown by the solid lines and the 95% confidence interval by the dashed lines.

Random forest feature selection was used for identifying important features of age-related changes based on the eye lens texture in CT. First, random forest-based feature selection was performed only on the control cohort, including patients with no hypertension and Fazekas score ≤1. This led to the identification of “Total Energy”, “RunPercentage”, “RobustMeanAbsoluteDeviation”, “GrayLevelNonUniformity”, “Idm”, “DifferenceAverage”, “Contrast”, “DifferenceVariance”, “LargeDependenceEmphasis”, “RootMeanSquared”, “DifferenceEntropy”, “Id”, “MeanAbsoluteDeviation”, “Uniformity”, “InverseVariance”, “Entropy”, and “Variance” as a feature set associated with age-related changes in the lens (Figure 3). Pearson’s correlation coefficient (r) was calculated to further evaluate the association between age and these radiomics features and was positively correlated with “Total Energy”, “GrayLevelNonUniformity”, “Idm”, “LargeDependenceEmphasis”, “RootMeanSquared”, “Id”, “Uniformity” (r = 0.39, 0.34, 0.48, 0.53, 0.48, 0.48, 0.46, respectively) and negatively correlated with “RunPercentage”, “RobustMeanAbsoluteDeviation”, “DifferenceAverage”, “Contrast”, “DifferenceVariance”, “DifferenceEntropy”, “MeanAbsoluteDeviation”, “InverseVariance”, “Entropy” as well as “Variance” (r = −0.52, −0.48, −0.48, −0.46, −0.45, −0.44, −0.51, −0.49, −0.52 and −0.47, respectively), (Figure 4).

### 3.3. Effect of Fazekas and Hypertension on the Eye Lens Texture

**Figure 5:**
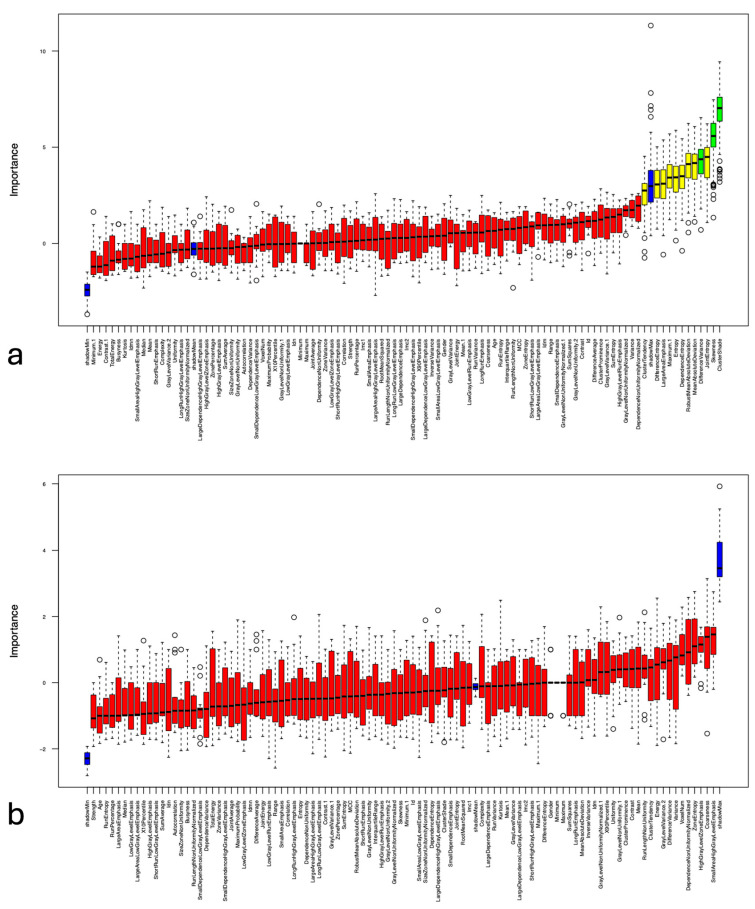
Random forest feature selection run by Boruta algorithm of the eye lens texture for differentiation between patients with a Fazekas score > 1 (**a**) or hypertension (**b**) and an age-matched control group. Confirmed features are shown in green, tentative features in yellow, features that were rejected in red and “shadow” features are displayed in blue. Circles represent outliers, while the whiskers denote the minimum and maximum values.

**Figure 6:**
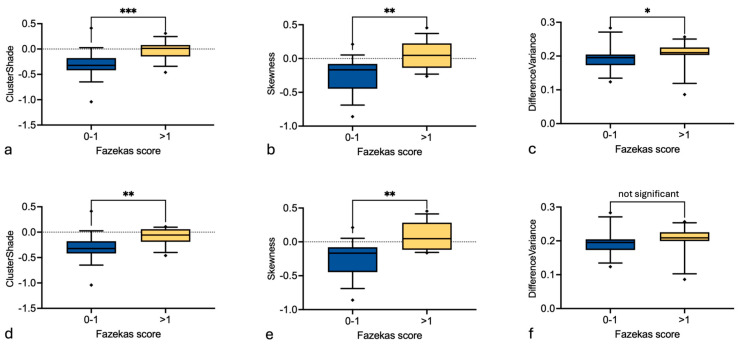
Boxplots of the three confirmed radiomic features to compare between patients with a Fazekas score 0–1 (blue) and >1 (yellow), (**a**): ClusterShade, (**b**): Skewness, (**c**): DifferenceVariance, as well as after exclusion of the overlapping group with both FAZEKAS > 1 and hypertension (lower row, (**d**–**f**)). *p* ≤ 0.05 is described as *, *p* ≤ 0.01 as ** and *p* ≤ 0.001 as ***.

Random forest feature selection of the eye lens texture in CT was used for differentiation between patients with a Fazekas score > 1 or hypertension and the control group, which was age-matched to account for age as a confounding variable. No confirmed features were found for the eye lens comparing hypertensive patients and control group. “ClusterShade”, “Skewness” and “DifferenceVariance” were confirmed to be relevant features to discriminate between patients with a Fazekas score > 1 and control group (Figure 5). Using the Mann–Whitney U test, we found significant higher values (*p* = 0.0006, 0.0023 and 0.0376, respectively) comparing patients with a Fazekas score > 1 versus ≤ 1 regarding all three features (“ClusterShade”, “Skewness” and “DifferenceVariance”, respectively). After exclusion of the overlapping group of four patients with both Fazekas score >1 and hypertension, “ClusterShade” and “Skewness” remained significant (*p* = 0.0045 and 0.0017) (Figure 6).

## 4. Discussion

To our knowledge, this study is the first CT-based radiomic analysis of the eye lens to detect differences among demographic or clinical groups with cerebral white matter lesions. We found that a set of 17 different CT-based radiomic features was associated with age-related changes in the eye lens. Furthermore, three different radiomic features of the eye lens were found to discriminate between age-matched patients with smaller versus larger WML volumes (Fazekas score ≤ 1 versus >1). Notably, no significant difference was found in radiomic features of the eye lens between age-matched patients with and without hypertension.

Radiomics has emerged as a powerful approach for quantitative analysis of medical imaging data [17]. Despite its growing use across multiple imaging domains, adoption within ophthalmology has remained limited. This may be attributed to the need for disease-specific analytical strategies, the technical complexity of ocular imaging, and the diversity of imaging modalities used in eye-related disorders [18,19]. Emergency department encounters, however, offer a unique setting for incidental identification of comorbid chronic pathologies, including cataracts, particularly given that a variety of individuals rely on emergency services in place of routine primary care [20]. Computed tomography is among the most frequently performed imaging examinations in emergency settings and has demonstrated utility in the assessment of traumatic cataracts [21]. However, there seems to be a scarcity of radiologic literature describing the CT appearance of age-related cataracts, which are much more prevalent than traumatic cataracts, as well as characteristics of the crystalline eye lens on CT imaging across different demographic populations [2]. One possible explanation is that the crystalline lens is a small and largely uniform structure on CT images and therefore tends to receive limited attention unless obvious abnormalities such as displacement, deformation, or absence are present. Nevertheless, the lens is consistently visualized on orbital CT and is frequently captured incidentally on routine head CT examinations, offering an underused opportunity for radiologic assessment of lens-related pathology [2]. Moreover, due to its regular geometry, consistent dimensions, and clear contrast with the surrounding aqueous and vitreous compartments, the lens represents a favorable candidate for automated or AI-driven segmentation approaches [22]. Therefore, an ultimate vision would be a radiomics-augmented radiology report in which validated biomarkers of the eye lens complement the conventional findings and lead to a model building through AI for clinical decision support. Although automated segmentation, which defines the spatial region from which radiomic features are extracted, has shown to be promising in increasing reproducibility [23], the clinical application of radiomics still faces challenges regarding standardization, reproducibility, Picture Archiving and Communication System/Radiology Information System integration and interpretability [24].

With aging, new fibroblasts are continually produced in the eye lens, which exert pressure on the older cells, causing them to shift inward from the outer pole toward the equator [25]. As a result, the older, denser fiber cells accumulate in the central region [26], and are exposed for a longer period to oxidative damage [27]. Furthermore, with advancing age, Glutathione, a powerful antioxidant, decreases in the eye lens, which results in protein aggregation, light scattering and age-related cataracts [28]. These age-related changes in the eye lens most probably contribute to the significant correlation of age and a set of 17 radiomics features of the eye lens texture, which we identified in this study. These radiomic biomarkers representing age-related changes in eye lens composition might be useful as potential new imaging biomarkers of eye lens function and probably age-related cataracts in future studies.

Oxidative stress plays a central role in the pathogenesis of age-related cataracts, and is also a key contributor to the development of atherosclerosis, primarily due to chronic inflammation associated with oxidative stress within the blood vessels [29]. This shared mechanism may help explain previously reported associations between cataract formation and cardiovascular disease [6,7,8]. White matter lesions, likely stemming from vascular causes, are among the most common age-related changes seen on brain MRI and are generally regarded as imaging markers of cerebral small vessel disease [30]. It is assumed that cerebral small vessel disease and atherosclerosis arise from overlapping risk factors [31] with vascular white matter lesions in older patients resulting from long-standing reductions in cerebral perfusion caused by lipohyalinosis, arteriosclerosis or fibrinoid necrosis of small vessels [32]. White matter lesions carry important clinical implications and have been linked to approximately a threefold elevation in dementia risk, as well as a twofold increase in both stroke incidence and mortality [33]. In addition, lesions of vascular origin are estimated to contribute to nearly 45% of dementia cases and around 20% of strokes, and are correlated with unfavorable neurological outcomes after stroke, including a higher likelihood of intracranial hemorrhage following mechanical thrombectomy [34,35]. In our study, a significant difference was found for radiomics features of the eye lens between age-matched patients with smaller versus larger WML volumes (Fazekas score 0–1 versus >1). All three identified features, “ClusterShade”, “Skewness”, and “DifferenceVariance” evaluate the disproportion of values about the mean value and are measures of heterogeneity [36]. They were significantly higher in patients with a Fazekas score > 1 compared to controls with a Fazekas score 0–1, thereby yielding a more heterogeneous picture of the eye lens in patients with higher white matter lesion load. Interestingly, previous studies found significantly reduced choriocapillaris perfusion, lower peripapillary as well as retinal vessel density in participants with WMLs when compared with healthy controls [37,38]. In contrast, the eye lens is avascular to remain clear for light transmission and relies on the surrounding aqueous humor as a blood surrogate. Studies have shown that oxygen delivery by the ciliary circulation seems to be the critical factor to sustain aqueous humor production and that hypoxia significantly reduced aqueous humor flow [39,40]. Microvascular changes leading to decreased oxygen delivery to the aqueous humor, might therefore result in loss of its function, which includes removing excretory products from metabolism, providing nutrition, transporting neurotransmitters, stabilizing the lens structure and regulating homeostasis [41]. Insufficient aqueous humor production might, in turn, lead to microstructural changes in the eye lens, which could be expressed as changes in textural parameters by radiomics analysis. However, it has to be stated that these interpretations remain hypothetical and require correlation with histopathology or advanced ophthalmologic imaging.

To the best of our knowledge, this is the first study to investigate the link between WML and CT-based changes in the crystalline eye lens. The positive relation between WML volume and heterogeneity of the eye lens in CT could be due to similar pathogenic factors, most probably vascular related. Although the eye lens is an avascular structure, the surrounding aqueous humor functions as a blood surrogate, which itself receives bloody supply from the ophthalmic artery. The ophthalmic artery constitutes the primary arterial blood supply to the eye and originates from branches of the internal carotid artery also perfusing the brain [39]. Because of this connection, as well as atherosclerosis being a systemic disease [42], pathological processes of orbital vascularization might mirror those of the central nervous system, including cerebral small vessel disease. CT-based radiomics analysis of the eye lens might therefore provide an opportunity for incidental discovery of comorbidities in patients visiting the emergency department and receiving orbital CT. To what extent this could aid in the diagnosis, evaluation, and follow-up of WML, cataracts as well as other lens pathologies, however, needs to be investigated in further studies.

This study has a number of limitations. To our surprise, no significant influence was found for hypertension on radiomic features of the eye lens in our study, although harmful, vascular related effects of hypertension on the eye has been reported [43]. This could be due to the limitation that we did not account for antihypertensive therapy as well as disease duration. Unfortunately, due to the retrospective study design, this information was not available to us in the electronic patient records. Another limitation is the small sample size of patients with hypertension and patients with a Fazekas score > 1, as well as overlaps between both groups, which may influence subgroup comparisons. Also, we did not adjust for other confounding factors, which potentially influence eye lens texture as well as microvascular health, such as cataracts, diabetes and metabolic abnormalities. Furthermore, differentiating precisely between the lens cortex and nucleus with CT is challenging because of the small size and potential volume averaging with neighboring structures. Nonetheless, any cortical alterations above the nucleus, or changes within the nucleus itself, would have been captured within the volume of interest if present. Additional limitations are inherent in the retrospective and single-center design of this study. Due to the limitation of being a small-scale pilot study with the risk of overfitting and false-positive findings, there is a need for further investigations with external validation, cross-validation, and correction for multiple testing. Expanded CT datasets compiled from multiple institutions could also allow the development and training of deep learning algorithms to predict pathologies of the eye lens.

## 5. Conclusions

As far as we are aware, this is the first study to perform a CT-based radiomic analysis of the eye lens to detect differences among demographic or clinical groups with cerebral white matter lesions. Performing radiomics analysis of the eye lens and by that expanding its scope in ophthalmology could contribute to a broader understanding of both lens physiology and lens-related diseases. The present results therefore expand the range of applications of radiomics and suggest a possible new biomarker for systemic vascular diseases.

## Figures and Tables

**Table 1:** Patient group characteristics.

Characteristics	Control Cohort	Fazekas Cohort	Hypertension Cohort	All Subjects
Number	82	17	17	112
Age (years) mean ± SD	41.2 ± 17.9	71.5 ± 13.0	59.6 ± 16.1	48 ± 20
Female *n* (%)	31 (37.8%)	7 (41.2%)	7 (41.2%)	43 (38.4%)
Indication for CT:				
Trauma	78 (95.1%)	15 (88.2%)	16 (94.1%)	105 (93.8%)
Foreign body	2 (2.4%)	0 (0.0%)	0 (0.0%)	2 (1.8%)
Surgery planning	1 (1.2%)	0 (0.0%)	0 (0.0%)	1 (0.9%)
Inflammatory focus	0 (0.0%)	1 (5.9%)	0 (0.0%)	1 (0.9%)
Postoperative control	1 (1.2%)	0 (0.0%)	1 (5.9%)	2 (1.8%)
Retrobulbar mass	0 (0.0%)	1 (5.9%)	0 (0.0%)	1 (0.9%)

## Data Availability

The datasets used and analyzed during this study are available from the corresponding author on reasonable request.

## Abbreviations

CT	Computed tomography
WML	White matter lesions
